# CAPPS: Congestion-aware payment and punishment scheme to stimulate selfish nodes in IoT-based VDTNs

**DOI:** 10.1371/journal.pone.0317107

**Published:** 2025-03-25

**Authors:** Ghani Ur Rehman, Ali Daud, Omar Ibrahim Aboulola, Bader Alshemaimri, Raed Alsini, Sajid Ullah Khan

**Affiliations:** 1 Department of Computer Science and Bioinformatics, Khushal Khan Khattak University, Karak, Pakistan; 2 Faculty of Resilience, Rabdan Academy, Abu Dhabi, United Arab Emirates; 3 Department of Information Systems and Technology, Collage of Computer Science and Engineering, University of Jeddah, Jeddah, Saudi Arabia; 4 Software Engineering Department, College of Computing and Information Sciences, King Saud University, Riyadh, Saudi Arabia; 5 Department of Information Systems, Faculty of Computing and Information Technology, King Abdulaziz University, Jeddah, Saudi Arabia; 6 Department of Information Systems, College of Computer Engineering and Sciences, Prince Sattam bin Abdulaziz University, Alkharj, Saudi Arabia; SASTRA Deemed University, INDIA

## Abstract

The Internet of Things (IoT) is facilitating the connection, identification, sensing, and analysis capabilities of digital devices; enabling them to perform tasks over the internet. Among various IoT applications, Vehicle Delay-Tolerant Networks (VDTNs) stand out, particularly in densely populated cities and rural areas with poor connectivity, where frequent network partitioning, unpredictable connections, and significant delays are common due to node selfishness. Where VDTNs offer promising solutions for such environments, they face challenges like congestion and selfish behavior due to big data traffic and limited resources. Existing systems aiming to mitigate selfishness often exacerbate congestion issues, necessitating a solution to address both concerns effectively. This article proposes the Congestion Aware Payment and Punishment Scheme (CAPPS) to address this issue. It incentivizes cooperation among vehicles and discourages selfish behavior by monitoring nodes. It achieves this by punishing nodes that intentionally discard messages and rewarding those successfully forwarding them. Additionally, CAPPS employs a load-sharing scheme to efficiently manage congestion, routing messages to less congested nodes instead of dropping them. The simulation results show the efficiency of the proposed approach over existing schemes, demonstrating enhanced performance. It improved the packet delivery ratio by around 24%, delivery delay by about 15%, and energy consumption by roughly 13%.

## 1 Introduction

The notion of IoT had originally come a decade before as a global network to increase the capabilities of 5G and other next-generation wireless communications technologies [1,2]. It is regarded as a promising topic of research because it could eventually be able to establish smarter and more intelligent communication across both physical and virtual nodes. According to the IoT idea, objects can act as nodes that can collect, process, store, and send data through different network component layers in a cooperative way of communication [3,4]. A de facto system of all smart, intelligent, ubiquitous, and pervasive applications has evolved as a result of many different technologies being combined and diverse communication protocols, and this evolution also affected the basic concept of the IoT [5–7]. IoT nodes connected to different sub-networks, such as WSN, MANET, or VANET, are part of the ecosystem. These sub-networks connect to gateways, which forward data to access points, more advanced levels of edge servers, and ultimately the cloud [8,9].

DTNs describe a specific kind of ad-hoc networks that have typically low-density levels, leading to intermittent communication between nodes [10,11]. For many applications like those described in [12], the DTN architecture is an acceptable approach for collaboration. DTN routing is based on the SCF concept that calls for storing bundles, or packets of data, inside the memory caches of the host node for a longer duration of time in the case that more efficient relay nodes are not available to forward bundles. The bundle is stored until a minimum of one better relay node opportunity presents itself. Following that, the bundles are either replicated or transmitted to the SCF node following the DTN protocol’s established routing strategy.

Vehicular DTNs (VDTNs), which are observed in the inconsistent routing situations that describe various vehicular mobility scenarios, are the result of the extension of DTN routing to Vehicular Networks (VANETs). In order to better manage barriers to vehicle communication, including radio obstacles, traffic signals, predefined mobility direction, and high speeds, VDTNs are more in need of the SCF principle than DTNs. The latency and reliability of the vehicular network are enhanced by the deployment of DTN [13]. The DTN functions can be applied to the vehicle environment by the VDTN’s class of networks. One of the most popular technologies for data flooding and packet replication to guarantee packet delivery is VDTNs, which is appropriate for prolonged delays, random and irregular connectivity, the lack of a central authority, and interoperability among complex networks. Data transmission in a difficult environment is made safe and effective by the VDTNs. These kinds of networks are infrastructure-less, rapidly arising networks that allow for packet flooding or replication to ensure packet delivery. For emergency evacuation scenarios where other traditional networks fail, VDTN is the perfect solution. Every vehicle in a VDTN acts as a router, and nodes working together to transmit packets is essential to the network’s seamless operation [14]. In a perfect environment, every node could assist forward packets and have sufficient resources to accomplish this. There are restricted resources available to vehicles in VDTNs, including buffer, computing power, energy, and bandwidth [15–17]. In order to protect these resources (free riders), particularly energy and buffer, nodes regularly dropped packets, refused to store them, or participated in self-serving data forwarding, which is for malicious purposes [18,19]. However, when a lot of messages are sent or received as a result of data traffic from VDTN networks, network buffer overflows, and messages are dropped, congestion on the buffer in the VDTN’s is likely to happen [20].

There are many frameworks developed such as Congestion Aware and Selfishness Aware Social Routing Protocol (CASASR) [21] and Credit-Based Congestion-Aware Incentive Scheme (CBCAIS) [22] to tackle the key problem of congestion and selfishness for DTNs. In CASASR, a more suitable relay node is selected for packet forwarding based on social attributes, a propensity for selfishness, and the buffer’s congestion status. In CBCAIS, to prevent relaying nodes from maliciously rejecting packets of data, a detection and punishing system is presented. However, these schemes have issues like (i) packets sent to congested nodes are discarded deliberately, (ii): No load-sharing mechanism is used, (iii): No proper monitoring of nodes. Nodes are encouraged for cooperation in a different way under the proposed strategy. Our proposed scheme has a novel approach to examine how the nodes are behaving that are causing congestion and not sharing the load for improved networking efficiency. The proposed scheme has monitoring nodes that will monitor the congestion and load sharing of the nodes. Some of the nodes deliberately discard the packets and do not share the load. The monitor nodes will identify all the nodes with such behavior. The nodes will be offered incentives in the form of payment. The node will be stimulated to share the load and hence congestion created by such nodes is marginally reduced. This improves the performance of the network. If the node repeatedly discards the packets deliberately, the node will be punished for such repeated behavior. The simulation’s findings show the proposed approach’s greater effectiveness compared to current strategies, demonstrating enhanced performance considering many different parameters including energy usage, packet delivery probability, and packet delivery delay. It improved the packet delivery ratio by around 24%, delivery delay by about 15%, and energy consumption by roughly 13%. The key contributions of this article are as follows:

- Investigate the challenges of selfishness and congestion in IoT-based VTDNs.
- In order to prevent vehicles from behaving selfishly, the Congestion Alert Payment and Punishment Scheme (CAPPS) and a load-sharing method have been proposed for employing in IoT-based VDTN’s.
- In order to carry out a comparison analysis, the outcome of the proposed system is compared to existing cutting-edge frameworks in terms of energy consumption, packet delivery delay, and packet delivery ratio.

The rest of this paper is organized as follows. Section 2 provides a detailed overview of different strategies regarding cooperation and congestion. The architecture of the proposed scheme is discussed in Sect 3. In Sect 4, the proposed framework CAPPS is discussed. The simulation setup and performance metrics have been discussed in Sect 5. The simulation and results are discussed in Sect 6. Sect 7 summarized the paper with a review of findings and recommendations for future studies.

## 2 Related work

Node availability has made it possible for VDTN’s to support urban inhabitants while addressing a particular urban challenge. The majority of options, however, need active involvement and cooperation from the nodes in the transportation, which doesn’t happen in reality. For instance, selfish nodes frequently only participate in the transfer of data with people or communities with whom they have a strong social connection or a shared interest. Other alternatives depend on centralized organizations or send too many redundant messages over the network. Therefore, to tackle the issue of selfishness and congestion in IoT-based VDTN’s, there are different approaches (Credit-Based, Reputation-Based, Game-theoretic, and Trust-Based) that are used to reduce congestion and encourage the selfish nodes to cooperate in the network.

### 2.1 Incentive-based approaches

Incentive schemes encourage the selfish nodes to work together to deliver data. The following are the incentives-based schemes that tackle the selfishness challenge.

Kou et al. [23] introduced a scheme called Detection and Punishment Selfishness (DPS) that provides an incentive-cooperation paradigm using a selfish routing algorithm and a punishment system, which stimulates nodes to cooperate and takes appropriate punitive actions when encountered with adverse nodal selfish behaviors. The strict incentive and punishment system between nodes, along with the monitoring system enhances efficient interaction between nodes. Aliedani et al. [24] proposed Deception Detection Mechanisms (DDMs) and illustrate their value in reducing the negative consequences of malicious vehicles. The work has broader implications for an open world of autonomous and adaptive systems with decentralized ownership and control that must collaborate to exploit shared resources. These systems are vulnerable to malicious behavior, thus they must be designed to be adaptable to it.

An Efficient Monitoring System (EMS) has been proposed by Rehman et al. [25] to identify non-cooperative nodes in networks. This scheme identifies and deals with selfish nodes to reduce the effectiveness of their entire network and data exchange. The strategy relies on cooperatively sharing node reputation across the entire network. Tracking misbehavior nodes is also essential to improve network performance generally. Sharma et al. [26] proposed a new scheme to implement selfish nodes in a DTN environment. The introduced scheme is based on reputation mode to detect selfish nodes in the network. Socievole et al. [27] introduced a social framework namely SORSI that controls the node selfishness in opportunistic communications. In order to maximize message distribution and a selfishness detection mechanism, SORSI takes advantage of the social-based nature of node mobility and other social properties of nodes. This is done to reduce selfish behavior and increase data forwarding. To incentivize vehicles to become involved in fog computing-based data forwarding in SIoVs, Xia et al. [28] presented a 2-D behavior-marker-based incentive scheme. First, they create a system for a 2-D behavior marker that may be utilized to fully assess vehicles. To address vehicular normal behavior, selfish behavior, and malevolent behavior, they also designed a currency credit-based data-forwarding incentive mechanism based on the 2-D marker and the social qualities of vehicle nodes.

Jiang et al. [22] proposed a scheme namely CBCAIS for DTNs to solve this issue. A detection and punishment system is suggested in CBCAIS to stop forwarding nodes from deliberately discarding packets. Rehman et al. [29] recommended a Socially omitting Selfishness Scheme (SOS) to encourage nodes to cooperate in SCC. In SOS, nodes are awarded payment for packet forwarding and at the same time when selfish behaviors are repeatedly displayed by nodes, they are punished. Rehman et al. [30] present a reliable community card system (RCCS) to address the issue of selfishness. It consists of two main components: a community-based card tracking system (CBCTS) and a reliable community. The CBCTS requires that each vehicle have a community card in order to be a part of the network, and the neighboring vehicles serve as monitoring vehicles. The reliable community is made up of all trustworthy vehicles with an appropriate degree of community reputation defined based on their honesty degree. Sharma et al. [31] proposed a reputation strategy with incentives to handle the issue of selfishness. The scheme clusters nodes based on social attributes computes the weighted social tie based on the strength of social connections and updates the weighted social tie by imposing a reward or penalty. If residual energy and packet delay have a trade-off or are penalized, then the incentive is given. To compute nodes’ reputation using social features, a new modularized deep nonnegative matrix deep autoencoder called IRU-mDANMF (incentivized reputation update by modularized DANMF) is developed.

### 2.2 Game-theoretic approaches

The non-cooperation nodes are identified and encouraged using a technique based on game theory. The game-theoretic methods discussed below have been proposed by different researchers to tackle the key problem of selfishness.

Rajput et al.[32] proposed a strategy called the Hunt game-based scheme, but it can only be fully operational when vehicles continue to be available and work together to build the network and offer services. However, the needs of the owner as well as the surrounding environment have a significant impact on the nodes’ behavior. As a result, nodes may leave the system at any time. A method for incentive-based collaboration is offered as well, which ensures that nodes will receive the best rewards possible based on their roles and levels of involvement in network activities. They have developed a static vehicular cloud testbed that offers Intelligent transportation services to vehicles, such as information on the most efficient routes, to evaluate the strategy. The authors in [33] introduced a novel method for identifying and punishing selfish behavior that they named DARWIN (Distributed and Adaptive Reputation mechanism for Wireless ad hoc Networks). It indicates that full collaboration may occur among nodes, and the scheme is also resistant to collusion. They also derive criteria under which no node can benefit from diverging from the framework. To tackle the issue of selfishness, Kanmani et al. [34] provide an efficient selfish node detection approach. In Game Theory with the Presence of Nash Equilibrium (NE), the Prices of Anarchy (PoA) and Stability (PoS) are examined for the Selfish Node Detection. In this experiment, we use PoA to identify selfish nodes in a network. Additionally, the Capacitated Selfish Resource Allocation (CSRA) game, which relies on dynamic least response, is presented to enhance resource utilization among nodes.

Nobahary et al. [35] proposed a scheme based on reputation and game theory for identifying selfish and malicious nodes. The proposed approach entails three stages: setup and clustering, data transmission and multiplayer game play, and update and malicious and selfish node detection. In the first step, the arrangements are made and clustering methods are performed. The nodes of each cluster cooperate in the second phase to play a continuously repeated game while transmitting packets of data from either their own or neighboring nodes. In the third phase, each node keeps track of how its neighbors are transmitting data packets, and the cooperative process is examined to identify the selfish or malicious nodes that delayed or failed to send the data packets. As a sort of punishment, the other nodes damage the reputation of the nodes who don’t work with them, and as revenge, they refuse to work with the self-centered and malicious nodes. Selfish and malicious nodes are therefore encouraged to work together. In order to discourage selfish behavior in Mobile Ad Hoc Networks and encourage selfish nodes to participate in network activities, Sharah et al. [36] proposed a slave mode selfish dynamic punishment scheme implementing cooperative repeated games. To render the penalized node exhausted and to stimulate it to collaborate with all nodes in the network, the strategy is utilized to apply a cooperative punishment from all nodes in the entire network. To identify inappropriate nodes in both active and passive attacks, Mostefa et al. [37] recommend a game theory-based method, specifically the Tit-For-Tat (TFT) strategy, as an acceptable paradigm to encourage human collaboration.

### 2.3 Trust-based approaches

Accurate evaluation of the nodes’ selfishness is also achieved through trust-based techniques. These are a few trust-based strategies that address selfishness.

To precisely evaluate the selfishness of the nodes, the authors in [38] presented a scheme called dynamic trust-based intrusion detection technique. The methodology takes into account both the direct trust level, which is based on direct communication interactions, and the indirect (recommended) trust level, which is based on solutions from their neighbors. Based on bitcoin, Park et al. [39] developed a secure and effective reward system for cooperative vehicular delay-tolerant communication networks. Although the well-known cryptocurrency and digital payment system Bitcoin makes use of cryptographic methods in its deployment, it is possible to construct a feasible credit-based reward system on vehicular networks at a modest cost. They also use scripts for Bitcoin transactions to manage their proposed incentive program. Nabais et al. [40] proposed a decentralized reputation strategy called BiRep, which identifies and penalizes Delay Tolerant-IoV black-hole nodes. For an IoV to function effectively, nodes must cooperate to some extent to send messages to their intended recipients. However, nodes could act inappropriately and secretly discard communications, a black-hole attack, which would harm network performance.

Delay-tolerant networks are used to address the problem of data congestion. For disaster areas, the authors in [41] developed a context-aware self-adaptive routing (CSRA) system in which each node is responsible for making independent judgments about data routing. The proposed protocol’s effects on energy, data delivery, and network overhead are assessed under various conditions. Nonetheless, this method emphasizes important messages about buffer congestion. Keykhaie et al. [42] presented an improved version of the socially aware congestion control algorithm (SACC), which builds its results based on the social attributes and congestion level of the node. The forwarding process is carried out using the social congestion meter (SCM), where messages are forwarded to nodes with greater SCM values. The node itself calculates the social connections between a node and the destination node in the event of congestion, and messages with the fewest or least social links are rejected rather than discarded at random. Abdi et al. [43] presented a multiphase approach based on game theory and direct and indirect reputation to encourage selfish and malicious nodes to collaborate in the Internet of Things. The strategy begins with the installation of nodes in the IoT network. To gather data, nodes are organized into clusters led by cluster heads during the initial phase. Then, in the second phase (multi-person game phase and data packet transmission), individuals engage in a dynamic, multi-person game where they promote their own or others’ data packets. In the following phase, the game’s outcome is utilized to impact nodes. In the third phase, nodes have the option to choose their data packet forwarding strategy (direct and indirect reputation update).

Liu et al. [21] proposed a social routing protocol called CASASR (congestion-aware and selfishness-aware social routing protocol). The recommended techniques choose a more suitable relay node based on social attributes, a propensity for selfishness, and the buffer’s congestion status. To choose a suitable relay node with a greater probability of meeting the deadline for sending a message within the remaining time-to-live message. Compared to several other modern algorithms, CASASR has outstanding outcomes in terms of reducing overhead and delivery ratio. Existing schemes have highlighted the issues related to the selfish behavior of nodes, congestion of traffic, and deliberate dropping of packets to save resources. Our proposed methodology has monitoring nodes to properly monitor every node in the network for data congestion, while the existing schemes focus on congestion control from source to destination only. This could lead to congestion of traffic on a single node, hence degrading the performance of the link in the network. The proposed methodology checks the load on the node, if the degree of the congestion is low in the node, it accepts it and forwards it to the neighbor nodes. The load is shared with the neighbor node if the node is experiencing a higher level of load. As a result, the network performs better overall. If the node deliberately drops the packets and is identified by the monitoring nodes, it will be punished to secure and optimize the resources of the network for better performance. A brief overview of the current incentive-based frameworks can be found in Table 1 in comparison with the proposed scheme.

**Table 1:** Summary of existing incentive-based frameworks in comparison with CAPP scheme.

Limitations of existing schemes	Advantages of CAPP scheme
Poor supervision of nodes that intentionally discard messages	Efficient monitoring system that keeps track of each node’s degree of congestion
The only contact with nodes it has a close social relationship	Every node taking part in the routing process has been given a similar priority
No proper strategy designed for load sharing with neighbor nodes in a situation of congestion	Proper Load-sharing scheme designed in case of congestion
No differences in incentives between full and no information	Incentives for full-state information differ significantly from those for no information.
Whenever the percentage of selfish nodes in the system rises, the overall efficiency of existing schemes deteriorates in terms of packet delivery probability, energy consumption, and delivery delay	The proposed approach performs efficiently in terms of packet delivery probability, energy usage, and delay in delivery of packets, whenever the system’s number of selfish nodes increases

## 3 System model

1. **Network model** In the network, a wide range of IoT nodes (vehicles) is assumed. The network is represented by N, which only has a minimal number of vehicles *V_i_*, where *i* = 1 , 2 , 3 , . . . , *n*. Eq 1 can be employed to denote the intended network.
  $$\begin{array}{c} N=\{V_{1},V_{2},V_{3},...,V_{i}\}. \end{array}$$ (1)
  Furthermore, it is presumed that every vehicle *V_i_* is provided with the capacity to carry out numerous network-related tasks as shown in Eq 2.
  $$\begin{array}{c} O=\{M_{n},p_{m},p_{n},\ell^{\prime }\}. \end{array}$$ (2)
  where, *M_n_* is the monitoring vehicle that constantly monitors the congestion degree, *p_m_* denotes payment awarded to different vehicles for their cooperation, *p_n_* is the node punishment for dropping the packets deliberately, and *ℓ^′^* represent the load sharing in case of high congestion.
2. **Node classification** There are three kinds of network nodes: monitoring nodes *M_n_*, cooperative nodes *C_n_*, and selfish nodes *S_n_*. The selfish nodes deliberately discard messages during the routing process. Cooperative nodes willingly forward messages to every other network node. The monitoring nodes keep a close eye on the degree of the congestion. Eqs 3–5 are used to define the node classification.
  $$\begin{array}{ccc} S_{n}\subseteq N & :=\{V_{i}\in (p_{n}\cup p_{m})\cap SD_{i}\} & \end{array}$$ (3)
  $$\begin{array}{ccc} C_{n}\subseteq N & :=\{V_{i}\in SD_{i}\} & \end{array}$$ (4)
  $$\begin{array}{ccc} M_{n}\subseteq N & :=\{V_{k}^{t}\in (p_{m}\cap \ell )\cup p_{n}\}. & \end{array}$$ (5)
3. **Design goal** When a buffer is limited, it’s important to convince selfish nodes that send messages to others, our goal is to design a Congestion Alert Payment and Punishment Scheme for IoT-Based VDTN. The approach explicitly aims to accomplish the following objectives:
  **1. Compatible with rewards**. In light of their selfish nature, all forwarding nodes adopt actual involvement as a result of our incentive mechanism, which can effectively encourage selfish nodes to deliver messages to various other nodes.
  **2. Awareness of congestion**. In addition to preventing deliberate message rejection by congested nodes, our technique can enhance node routing performance during congestion.
4. **Threshold calculations**
  The nodes are awarded payment for the successful forwarding of messages. At the same time, nodes can also be punished for deliberately dropping messages. Therefore, the threshold values for both *p_m_* and *p_n_* can be calculated by using Equations 6 and 7,
  $$\begin{array}{ccc} & T_{p_{m}}\in (0,\lambda )\in N:\{V_{i}\in p_{m}≼\lambda \in (0,1)\} & \end{array}$$ (6)
  $$\begin{array}{ccc} & T_{p_{n}}\in (\lambda ,1)\in N:\{V_{i}\in p_{n}≽\lambda \in (0,1)\} & \end{array}$$ (7)

## 4 Proposed congestion alert payment and punishment scheme (CAPPS)

**Fig 1:**
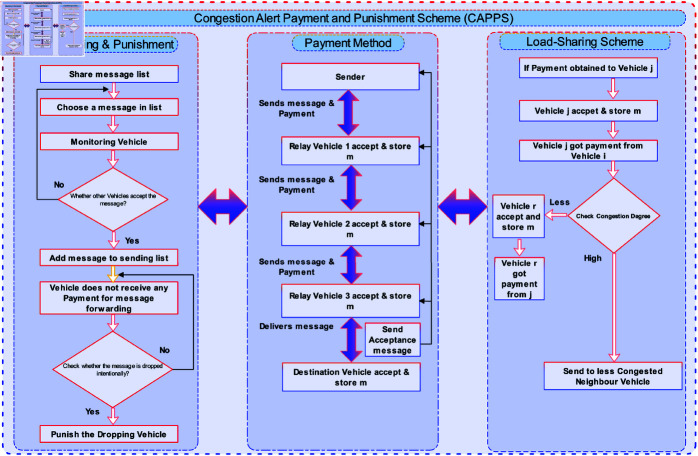
Overall structure of the proposed scheme.

The proposed scheme aims to ensure that all nodes in VDTN’s networks utilize their resources. It also reduces congestion by encouraging packet shifting as a solution to discarding it. Although most studies in the related works decide to tackle congestion by dropping packets, in our study, we tackle an emergency by moving older packets to less congested vehicles that suffer from a higher delivery delay and *TTL*. Hence, our technique is unique in that it guarantees that payment is awarded to each vehicle that takes part in the packet delivery process and enables the shifting of packets instead of dropping. The proposed framework is divided into three primary sections: monitoring and punishment mechanism, payment method, and load-sharing. The proposed scheme’s general structure is shown in Figure 1. The monitoring nodes properly keep an eye on the nodes’ behaviors during the Monitoring and Punishment phase of the proposed technique. When a node exhibits selfish behavior and intentionally drops packets, it stimulates packet forwarding by providing some kind of incentive. However, nodes that consistently behave selfishly can be punished. To motivate selfish vehicles to participate in the routing process, the acceptance-based payment approach is used in the second phase of the proposed scheme. It offers a reward for each network vehicle that delivers the message. The load-sharing technique has been developed to handle a wide range of possible congestion scenarios. When too many packets are received by a vehicle with a high connection degree, it becomes congested and eventually overflows its buffer. In other scenarios, it rejects packets in situations where overflow is predicted to happen or has already happened from adjacent vehicles. Furthermore, it monitors the node’s load; if it is not too congested, it accepts the load and forwards it to the neighboring node. If the node faces a higher degree of load, the load is shared with the neighboring node.

### 4.1 Monitoring and punishments scheme

**Fig 2:**
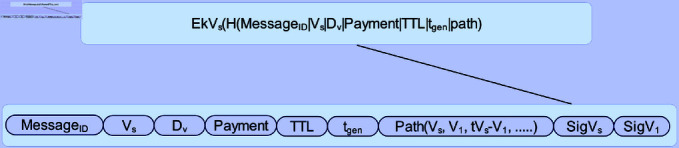
Forwarding ticket.

The purpose of the monitoring and punishment system is to ascertain whether any confirmed vehicles have disposed of messages intentionally. A vehicle can anticipate penalties if it has been deliberately not considering messages. The vehicle manifesting misbehavior has to be the final forwarding vehicle on the relaying path whenever a message is intentionally discarded. To investigate vehicles that exhibit inappropriate behavior, the monitoring vehicle has to know the message-forwarding route information. To do this, transmitting vehicle *V_s_* a forwarding ticket has been generated as shown in Fig 2, if it comes across a forwarding vehicle *V*_1_ that has a shorter delivery time.

The message identifier in the sending ticket is *Message_ID_*; the vehicle identifiers of the packet sending vehicle and receiving vehicle, are *V_s_* and *D_v_* respectively; Payment, as given in Eq 8, is the maximum number of payment to be paid for a message that is successfully delivered;


$$\begin{array}{c} RP_{m}(T^{D})=\{\begin{array}{cc} P_{m}(1-T^{\frac{D}{TTL}}),\; & if\;0<T^{D}<TTL\\ 0,\; & if\;T^{D}\geq TTL\\ \; \end{array} \end{array}$$ (8)

*RP_m_*(*T^D^*) is the total amount of payment that the relaying will receive from the destination vehicle based on the delivery delay *T^D^*. *TTL* is the message duration, and *p_m_* is the maximum number of credits that the destination node can pay for a message, assuming 1 in this design.

While the message creation time is *t_gen_*, the message duration is *TTL*. The path is the name of the message-sending path. The time interval between Vehicle *V_s_* and *V*_1_ is denoted by *tV_s_*−*V*1, and their digital signatures are *SigV_s_* and *SigV*_1_, respectively. The Vehicle *V_s_*’s private key is denoted by *Ek_Vs_*, and the hash function *H* is used to provide an overview that guarantees the message integrity.

Vehicles *V_s_* and *V*_1_ will retain a copy of the sending ticket as independent evidence of forwarding after it has been generated. Vehicle *V*_1_ will add its vehicle identification, duration of contact with the transmitting path, and authenticate data employing a private key to produce an extra forwarding ticket whenever it comes across another, *V*_2_, that has a less delivery delay. The process will be repeated by other forwarding vehicles until they come across the destination vehicle. It is evident that due to their digital signatures, vehicles are unable to alter the forwarding path or refuse forwarding behavior. Consequently, the message’s final forwarding vehicle has to be identified by the Trusted Third Party (TTP) utilizing ticket forwarding. TTP can not determine whether a message was lost intentionally or as a result of the *TTL* expiring, even though it can locate the last forwarding vehicle. The forwarding vehicle’s and the destination vehicle’s contact details must be provided for TTP to figure out whether or not a message was deliberately dropped. In order to make up for the loss of the other vehicle, some Payment will be deducted from the misbehaving vehicle. Since any normal vehicle would not intentionally discard messages, CAPPS assumes that the misbehaving vehicles would be punished by having many payments deducted from them.

### 4.2 Payment method

**Fig 3:**
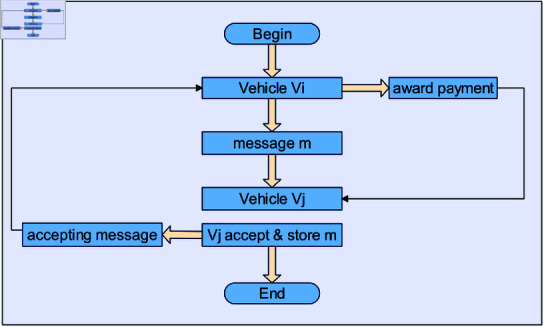
Flowchart for payment scheme based on message accepting.

**Fig 4:**
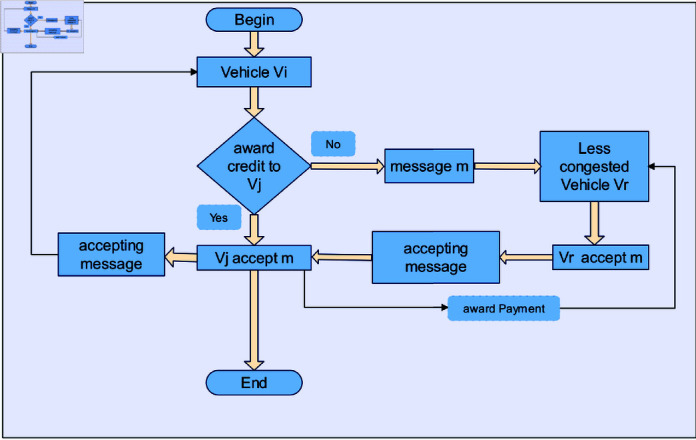
Flowchart for Load-sharing method to control congestion and sending a message to less congested vehicle.

The acceptance-based payment technique is used to motivate selfish vehicles to take part in the routing process. Every network vehicle that accepts the message will get some reward under the proposed system. The payment scheme based on message accepting is presented in Algorithm 1. The flowchart for payment scheme based on message accepting is shown in Fig 3. In Algorithm 1, the Vehicle *V_i_* is going to provide some rewards from its earned credits whenever Vehicle *V_i_* sends a packet to Vehicle *V_j_*, and Vehicle *V_j_* accepts the packet. After receiving an acceptance message that provides the vehicle with the buffer status information, payment will be issued. This buffer status information will demonstrate whether or not the node has accepted the message. By looking up the message’s *ID* in the buffer state data provided by the recipient, the source can verify that the packet was accepted and stored. Every vehicle will receive a reward or incentive from the destination vehicle upon successful delivery of a message. The flowchart for the Load-sharing method to control congestion and send messages to less congested Vehicles is shown in Fig 4.

**Algorithm 1**. Algorithm for payment scheme based on message accepting.

**INPUT:** Vehicles *V_i_* and Vehicle *V_j_*

**OUTPUT:** Payment to vehicle for message acceptance

1: Begin

2: *V_i_* send message *m* to *V_j_*

3: *V_j_* accept the message *m*

4: *V_j_* store message *m*

5: *V_j_* send an accepting message for *m*

6: *V_i_* awarded credit to *V_j_*

7: End

### 4.3 Load-sharing scheme

The load-sharing method was developed for many different scenarios in which congestion can occur. A high connection degree vehicle suffers congestion when it receives an excessive amount of packets, which causes a buffer overflow. In other situations, it rejects packets from nearby vehicles when overflow is either predicted to happen or has already happened. The third possibility is that although the vehicle itself is congested, so are the vehicles nearby. The vehicle can decide not to receive a packet in low-energy situations in order to maintain its resources.

The proposed strategy is to change the messages to address these types of circumstances. After verifying the level of congestion, messages will be accepted if the newly arrived vehicle provides more credit with lower TTL and delivery latency as well is given in Algorithm 2. Vehicle *V_j_* in Algorithm 2 accepts a packet from Vehicle *V_i_*. Once a packet is accepted, the Vehicle *V_i_* gives *V_j_* a reward. When the Vehicle *V_j_* noticed that its buffer remained full, it forwarded the packet to another, less congested Vehicle *V_r_*. Since the older message has a higher delivery delay and TTL, the more congested vehicle will get it first when a new message having greater reward arrives in Algorithm 3. The strategies of the load-sharing approach can be seen in Algorithm 4 for controlling traffic vehicles and their congested neighbors.

**Algorithm 2.** Load-sharing method to control congestion and sending a message to less congested vehicle.

**INPUT:** Number of vehicles

**OUTPUT:** Controlling Congestion

1: Begin

2: **if** *Credit_c_*←*V_j_* **then**

3:   *V_j_* accept message *m*

4:   *V_j_*←*m*

5:   *V_j_* send an accepting message *m*

6:   *V_j_*←*Credit_c_* from *V_i_*

7: **else**

8:   forward *m* to less congested Vehicle *V_r_*

9:   *V_r_* accept message *m*

10:   *V_r_*←*m*

11:   *V_r_* send an accepting message to *V_j_*

12:   *V_r_*←*Credit_c_* from *V_j_*

13:   *V_r_* accept message *m*

14: **end if**

15: End

**Algorithm 3.** Load-sharing method to control congestion and sending a message to Extremely congested vehicle.

**INPUT:** Number of vehicles

**OUTPUT:** Controlling highly Congested Vehicle *V_x_*

1: Begin

2: **if** message *m* is send to high congested Vehicle *V_x_*

3:  Examine the message that is the oldest in the buffer

4:  *TTL* ← *high*

5:  *Ddelay* ← *high*

6:  Send the oldest message to a neighbour Vehicle *n_v_* whose level of congestion is low

7: **else**

8:  Compute TTL

9:  Compute Delivery Delay

10:  **if** Payment is obtained **then**

11:   *Accept* ← *m*

12:   *Store* ← *m*

13:  **else**

14:   Forward *m*←*n_v_*

15:  **end if**

16: **end if**

17: End

**Algorithm 4.** Load-sharing method to control congestion when the neighbor vehicle and receiver vehicle are congested too.

**INPUT:** Number of vehicles

**OUTPUT:** Controlling Congestion for *n_v_*

1: Begin

2: **if** *V_i_* is congested **then**

3:  send *m*←*n_v_*

4:  **if** *n_v_*←*congested* **then**

5:   inform TTP about it.

6:   trip taken by TTP

7:   TTP transmits a message to a vehicle having minimal or no congestion

8:  **else**

9:   Initiate the message acceptance mechanism

10:  **end if**

11: **end if**

12: End

**Fig 5:**
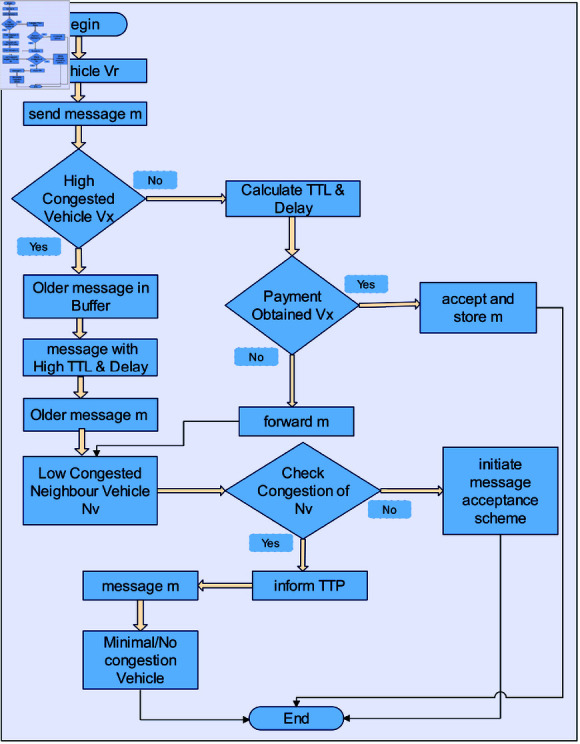
Flowchart for load-sharing method to control congestion and sending a message to extremely congested vehicle or when the neighbor vehicle and receiver vehicle are congested too.

In Algorithm 4, when a new packet arrives and TTP detects that its neighboring vehicles are also congested, it reports the congestion and sends data regarding the vehicle with minimal or no traffic to another vehicle with minimal congestion. The flowchart for the Load-sharing method to control congestion and sending a message to the extremely congested vehicles or when the neighbor vehicle and receiver vehicle are congested too is shown in Fig 5. The scheme CAPPS needs considerable challenges to be addressed when implementing it in the real world. The challenges need careful consideration to ensure the effective behaviors of nodes in VDTNs. As there are thousands of nodes across the roads like RSU, vehicles, and other nodes the nodes increase with time. The scheme needs to be scalable to accommodate the processing and communications overhead when making payments and punishments. Blockchain technology can be used to distribute the processing and communications overhead in a hierarchical manner. The cost to deploy the system needs devotion both in hardware and software to enhance its performance. The complexity of IoT-based VDTNs and integrating them with existing systems requires the compatibility of different protocols and standards. Integrating the protocols of VDTNs with current systems is a challenging task and may require middleware that can bridge between the current and VDTN systems. The standardization of protocols can also improve the interoperability issues to enhance the system’s performance.

## 5 Simulation setup

The VDTNsim simulator [44] was used to examine the performance of the presented cooperation methods. Each simulation runs six hours. There are 60 vehicles in all. The area used for simulation is 500*m* ∗ 1000*m*. Fixed/Vehicular Mobility is the type of mobility model utilized in simulation. The speed range for mobile nodes is 0 m/s to 15 m/s. The packet size and the packet rate are 100 bytes and 1 packet/s. There are 10, 20, 30, 40, and 50 cooperative nodes in the network. The buffer size for terminal nodes is 200 MB. IEEE 802.11b is the communication standard used in this article. Table 2 contains a list of the simulation’s parameters.

**Table 2:** Simulation parameters.

Parameter	Values
Simulation time	6 hrs
simulation area	500*m* ∗ 1000*m*
Total number of vehicles/nodes	60
No. of cooperative nodes	10,20,30, 40, 50
Number of terminal nodes	15
No. of relay nodes	15
Terminal nodes buffer capacity	200MB
Package size	100 bytes
Packet rate	1 Packet/sec
Relay nodes buffer capacity	300MB
Mobile node speed	0 m/s to 15 m/s
Routing protocols	Spray and wait, epidemic, and ProPhet
Mobile nodes buffer capacity	120 MB
Data throughput	10 Mbps
No. of bundles copies	N=02
Nodes communication standard	IEEE 802.11b

The generation and exchange of packages are simulated for a total of 6 hours in all of the experiments. The performance of Epidemic [45], ProPhet [46], and Spray-and-Wait [47] is evaluated for each scenario. The epidemic is a routing strategy that uses flooding to reproduce packages at each contact possibility. A vehicle can send as many copies as it has requested using this routing scheme. Spray-and-Wait, on the other hand, limits how many copies of each package can be made. The two parts of this routing protocol’s efficacy are “spray” and “wait.” During the “spray step,” R replicas of each original package are transmitted to R different relays. Throughout the “wait phase,” this communication routing method awaits the delivery of the data packet to the desired node by any of the R relays. The Spray-and-Wait variation that is binary is investigated in this study, in which the source node starts with N copies to be distributed with each package (four copies during the evaluation). Furthermore, at a particular node A with more than one bundle copy, it transfers the remaining copies to an additional node *N* = 2 package copies, retaining the remainder independently. When a node has just a single copy left to send, it transmits that copy to the ultimate destination and nothing else.

### 5.1 Performance metrics

For performance indicators in a network, the simulation employs package delivery Probability, average delivery delay, and average energy consumption. The successful transmission of messages over all of the generated messages is referred to as the packet delivery ratio. Average delivery delay is the time it takes for a message to reach its intended recipient. The duration of time required for a message to reach its ultimate recipient is known as the average delivery delay. Average energy refers to the ratio of individual vehicle energy usage to total node energy usage.

## 6 Simulation results and discussion

**Fig 6:**
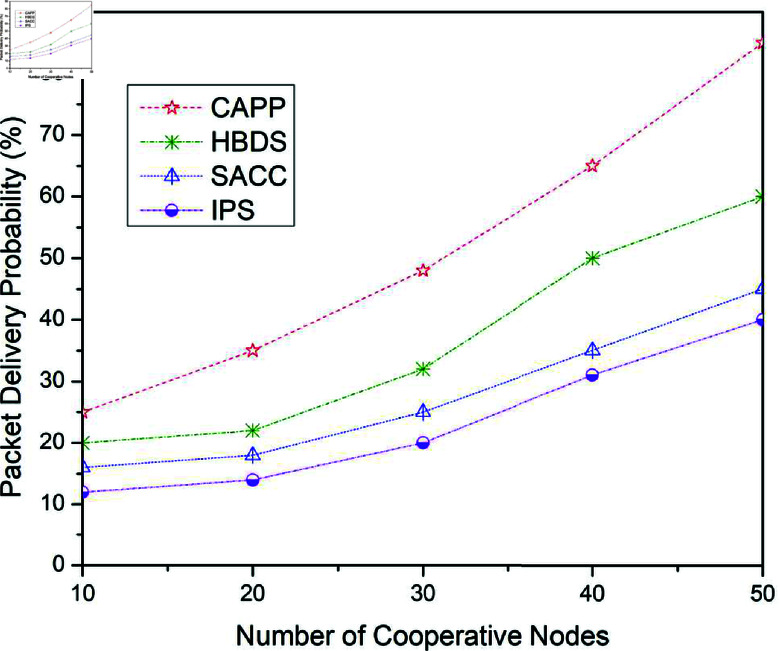
Package delivery probability for CAPPS, HBDS, SACC, and IPS Cooperation methods with Epidemic routing protocol as a function of the number of cooperative nodes.

**Fig 7:**
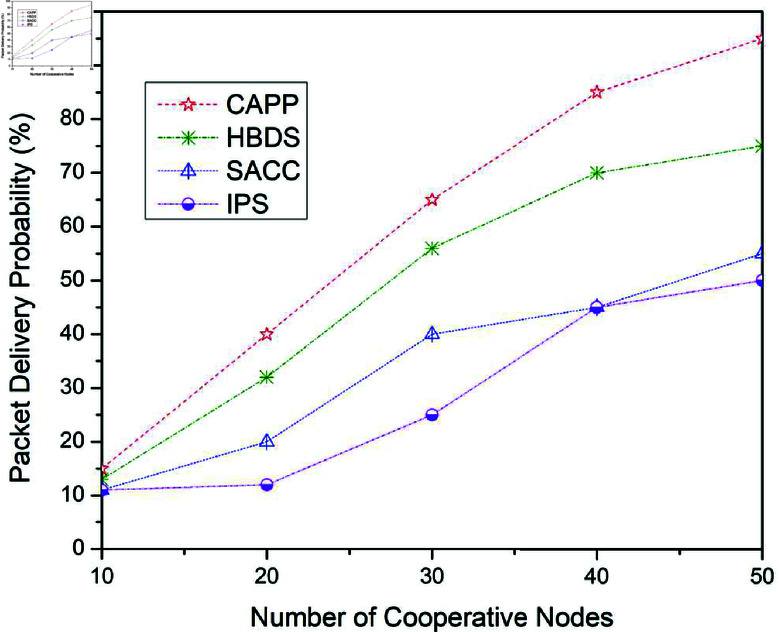
Package delivery probability for CAPPS, HBDS, SACC, and IPS Cooperation methods with ProPhet routing protocol according to the number of cooperative nodes.

**Fig 8:**
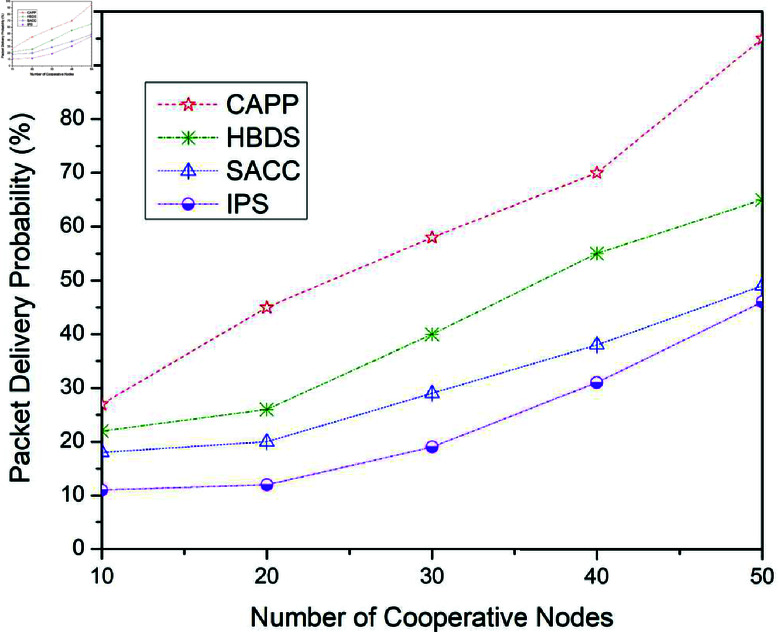
Packet delivery probability for CAPPS, HBDS, SACC, and IPS Cooperation methods with Spray and Wait routing protocol according to the number of cooperative nodes.

**Fig 9:**
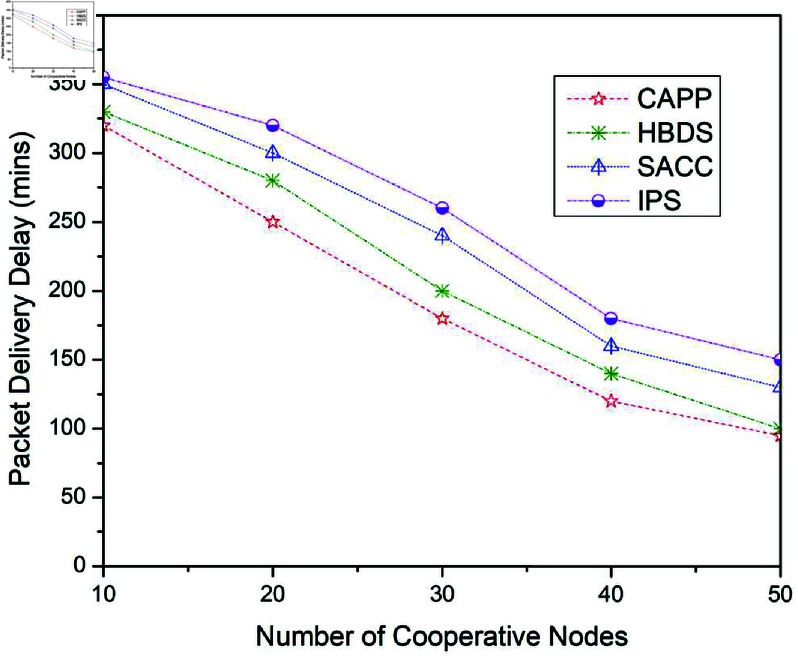
Packet delivery delay for CAPPS, HBDS, SACC, and IPS Cooperation methods with Epidemic routing protocol according to the number of cooperative nodes.

**Fig 10:**
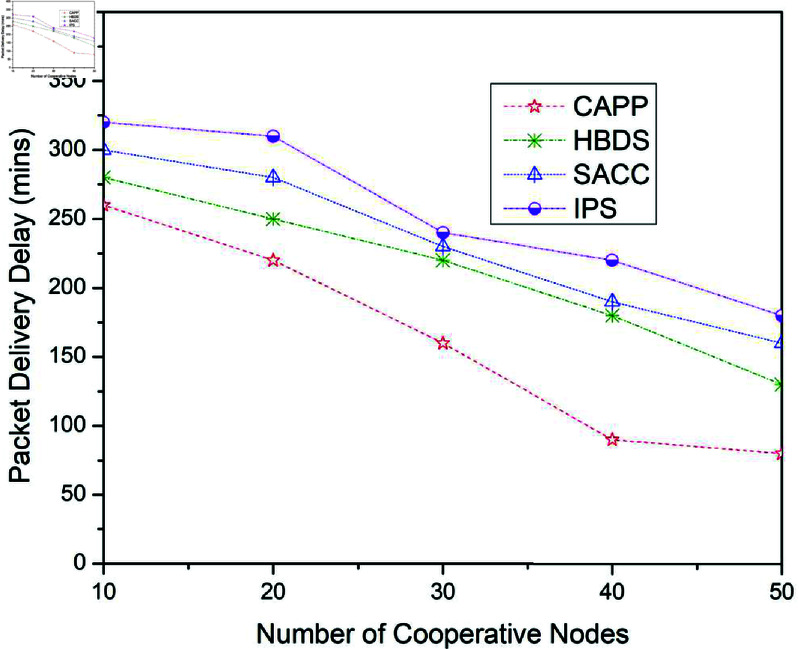
Packet delivery delay for CAPPS, HBDS, SACC, and IPS Cooperation methods with ProPhet routing protocol according to the number of cooperative nodes.

**Fig 11:**
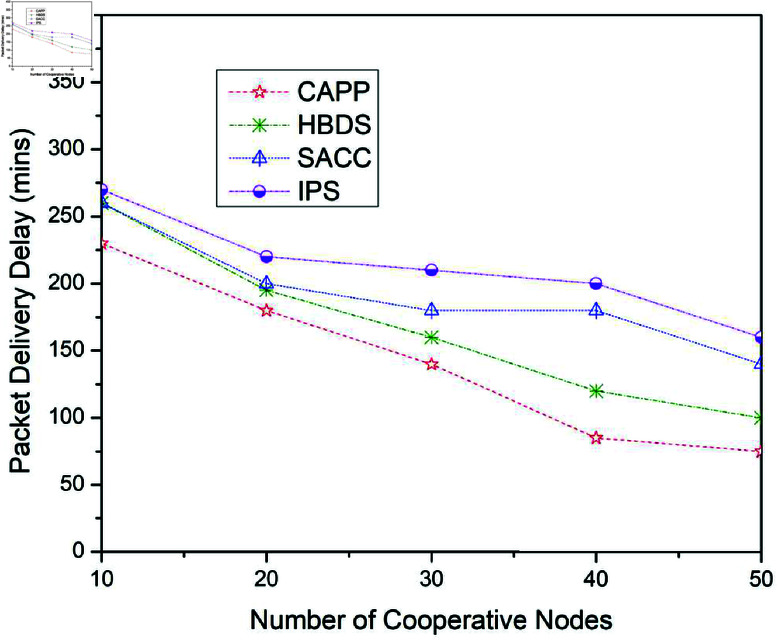
Packet delivery delay for CAPPS, HBDS, SACC, and IPS Cooperation methods with Spray and Wait routing protocol according to the number of cooperative nodes.

**Fig 12:**
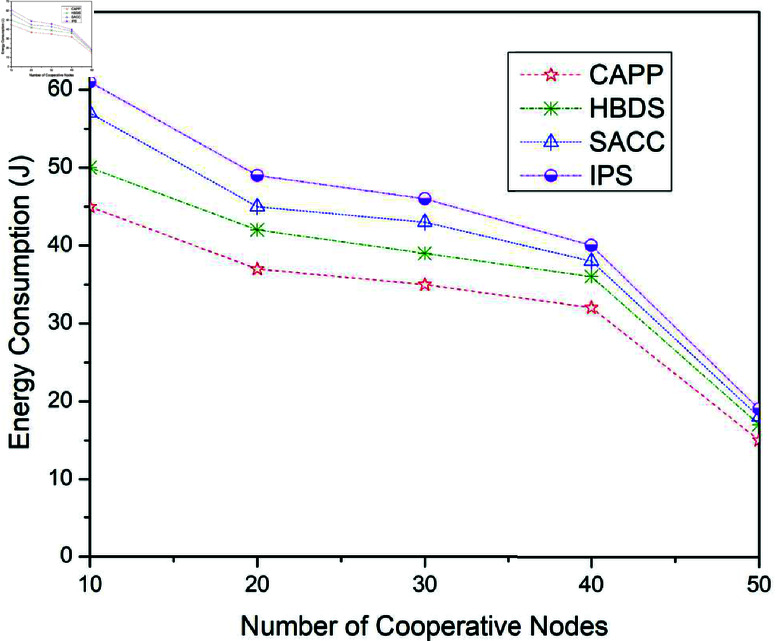
Energy consumption for CAPPS, HBDS, SACC, and IPS Cooperation methods with Epidemic routing protocol according to the number of cooperative nodes.

**Fig 13:**
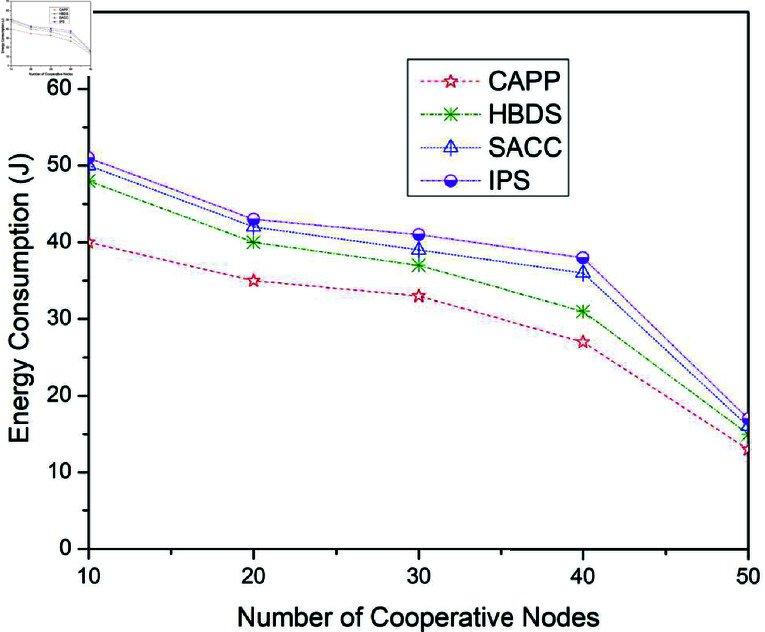
Energy consumption for CAPPS, HBDS, SACC, and IPS Cooperation methods with ProPhet routing protocol according to the number of cooperative nodes.

**Fig 14:**
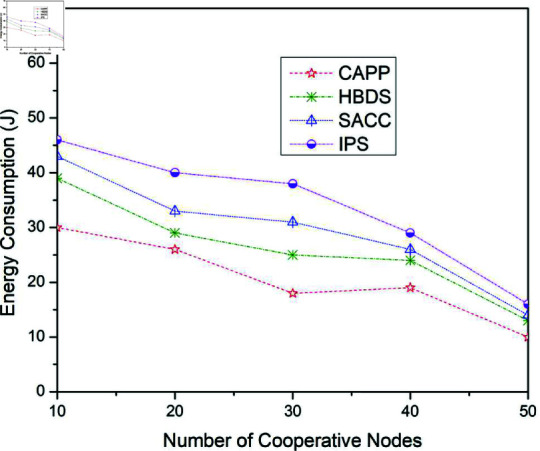
Energy consumption for CAPPS, HBDS, SACC, and IPS Cooperation methods with Spray and Wait routing protocol according to the number of cooperative nodes.

This part emphasizes the performance evaluation of the findings received from the previously mentioned scenario’s experiments. As illustrated in Figs 6 to 14, when four previously presented cooperation mechanisms are applied to the Epidemic, ProPhet, and Spray and Wait routing protocols. Fig 6 illustrates the outcomes for the package delivery probability when the Epidemic routing method is used with the four mentioned earlier cooperation strategies.

For 10 cooperative vehicles, the package delivery probability of CAPPS, HBDS, SACC, and IPS is 25%, 20%, 16%, and 12% respectively. It can be seen that the CAPPS technique outperforms the other strategies. When compared to the HBDS, SACC, and IPS schemes, it enhances entire network performance by about 6%, 10%, and 14% respectively. Additionally, for 30 cooperative vehicles, the package delivery probability of CAPPS, HBDS, SACC, and IPS is 48%, 32%, 25%, and 20% respectively. Again it can be seen that the CAPPS technique outperforms the other strategies. When compared to the HBDS, SACC, and IPS schemes, it enhances entire network performance by about 18%, 26%, and 31% respectively. These findings align with the basic flooding behavior of the Epidemic as well, which wastes network resources, but also with the approach network node collaboration is imposed. Again it can be seen that the CAPPS technique outperforms the other strategies. When compared to the HBDS, SACC, and IPS schemes, it enhances entire network performance by about 18%, 26%, and 31% respectively. These outcomes correspond not just to the Epidemic’s fundamental flooding behavior, which wastes network resources, but also with the approach network node collaboration is imposed.

Similarly, for 50 cooperative vehicles, the package delivery probability of CAPPS, HBDS, SACC, and IPS is 85%, 60%, 45%, and 40% respectively. Again the CAPPS technique outperforms all the compared techniques namely HBDS, SACC, and IPS schemes by around 27%, 44%, and 50% respectively. It is clearly illustrated in Fig 6, as the number of cooperating mobile nodes grows, so does the probability of a package being delivered. This is due to an increased number of interaction opportunities that happen during the simulation period, allowing nodes to send more packages.

Fig 7 illustrates the outcomes for the packet delivery probability when the Prophet routing method is used with the four previously mentioned cooperating strategies. For 10 cooperative vehicles, the package delivery probability of CAPPS, HBDS, SACC, and IPS is 15%, 13%, 11%, and 08% respectively. It can be seen that the CAPPS technique outperforms the other strategies. When compared to the HBDS, SACC, and IPS schemes, it enhances entire network performance by about 2%, 4%, and 7% respectively. Additionally, for 30 cooperative vehicles, the package delivery probability of CAPPS, HBDS, SACC, and IPS is 65%, 56%, 40%, and 25% respectively. Again it can be seen that the CAPPS technique outperforms the other strategies. When compared to the HBDS, SACC, and IPS schemes, it enhances entire network performance by about 9%, 25%, and 40% respectively. Similarly, for 50 cooperative vehicles, the package delivery probability of CAPPS, HBDS, SACC, and IPS is 95%, 75%, 55%, and 50% respectively. Again the CAPPS technique outperforms all the compared techniques namely HBDS, SACC, and IPS schemes by around 20%, 40%, and 45% respectively. The CAPPS method outperforms all of the compared schemes, including HBDS, SACC, and IPS, because they employ measures to get nodes to return to the protocol when they deviate from it. The packet delivery probability of CAPPS, HBDS, SACC, and IPS with Epidemic, Spray and Wait, and ProPhet routing protocol can be illustrated in Tables 3, 4 and 5 respectively.

Fig 8 illustrates the outcomes for package delivery probability when the four earlier stated cooperative mechanisms are applied to the Spray and Wait routing scheme. For 10 cooperative vehicles, the package delivery probability of CAPPS, HBDS, SACC, and IPS is 27%, 22%, 18%, and 11% respectively. It can be seen that the CAPPS technique outperforms the other strategies. When compared to the HBDS, SACC, and IPS schemes, it enhances entire network performance by about 5%, 9%, and 16% respectively. Additionally, for 30 cooperative vehicles, the package delivery probability of CAPPS, HBDS, SACC, and IPS is 58%, 40%, 29%, and 19% respectively. Again it can be seen that the CAPPS technique outperforms the other strategies. When compared to the HBDS, SACC, and IPS schemes, it enhances entire network performance by about 18%, 29%, and 39% respectively. Similarly, for 50 cooperative vehicles, the package delivery probability of CAPPS, HBDS, SACC, and IPS is 95%, 65%, 49%, and 46% respectively.

Again the CAPPS technique outperforms all the compared techniques namely HBDS, SACC, and IPS schemes by around 30%, 46%, and 49% respectively. In this routing protocol, the influence of the recommended cooperative strategy is a significantly more apparent way to improve network performance.

It can also be seen for package delivery delay in Figs 9 to 11, when the four previously presented cooperation mechanisms are applied to the Epidemic, ProPhet, and Spray and Wait routing protocols. Fig 9 illustrates the outcomes for the package delivery delay when the four previously stated cooperative mechanisms are applied to the Epidemic routing scheme. For 10 cooperative vehicles, the package delivery delay of CAPPS, HBDS, SACC, and IPS is 320%, 330%, 350%, and 355% respectively. It can be seen that the CAPPS technique outperforms the other strategies. The delivery delay of the CAPPS scheme is approximately 2%, 8%, and 9% lower than the HBDS, SACC, and IPS schemes respectively. The packet delivery delay of CAPPS, HBDS, SACC, and IPS with Epidemic, Spray and Wait, and ProPhet routing protocol can also be seen in Tables 6, 7, and 8 respectively.

**Table 3:** Package delivery probability for CAPPS, HBDS, SACC, and IPS with epidemic routing protocol according to the number of cooperative nodes.

No. of cooperative nodes	CAPPS	HBDS	SACC	IPS
10	25	20	16	12
20	35	22	18	14
30	48	32	25	20
40	65	50	35	31
50	85	60	45	40

**Table 4:** Package delivery probability for CAPPS, HBDS, SACC, and IPS with Spray and Wait routing protocol according to the number of cooperative nodes.

No. of cooperative nodes	CAPPS	HBDS	SACC	IPS
10	27	22	18	11
20	45	26	20	12
30	58	40	29	19
40	70	55	38	31
50	95	65	49	46

**Table 5:** Package delivery probability for CAPPS, HBDS, SACC, and IPS with ProPhet routing protocol according to the number of cooperative nodes.

No. of cooperative nodes	CAPPS	HBDS	SACC	IPS
10	15	13	11	11
20	40	32	20	12
30	65	56	40	25
40	85	70	45	50

**Table 6:** Packet delivery delay for CAPPS, HBDS, SACC, and IPS with Epidemic routing protocol according to the number of cooperative nodes.

No. of cooperative nodes	CAPPS	HBDS	SACC	IPS
10	320	330	350	355
20	250	280	300	320
30	180	200	240	260
40	120	140	160	180
50	95	100	130	150

**Table 7:** Packet delivery delay for CAPPS, HBDS, SACC, and IPS with Spray and Wait routing protocol according to the number of cooperative nodes.

No. of Cooperative Nodes	CAPPS	HBDS	SACC	IPS
10	230	260	260	270
20	180	195	200	220
30	140	160	180	210
40	85	120	180	200
50	75	100	140	160

**Table 8:** Packet delivery delay for CAPPS, HBDS, SACC, and IPS with ProPhet routing protocol according to the number of cooperative nodes.

No. of Cooperative Nodes	CAPPS	HBDS	SACC	IPS
10	260	280	300	320
20	220	250	280	310
30	160	220	230	240
40	90	180	190	220
50	80	130	160	180

Additionally, for 30 cooperative vehicles, package delivery delay of CAPPS, HBDS, SACC, and IPS is 180%, 220%, 270%, and 280% respectively. Again it can be seen that the CAPPS technique outperforms the other strategies. The delivery delay of the CAPPS scheme is 10%, 22%, and 25% lower than HBDS, SACC, and IPS schemes respectively. Similarly, for 50 cooperative vehicles, the Delivery delay of CAPPS, HBDS, SACC, and IPS is 110%, 130%, 170%, and 180% respectively. Again the CAPPS technique outperforms all the compared techniques namely HBDS, SACC, and IPS schemes by around 5%, 15%, and 18% respectively. The CAPPS method outperforms all of the compared schemes, including HBDS, SACC, and IPS. The package delivery delay begins to reduce as more opportunities for contact arise, as seen in Fig 9.

Fig 10 illustrates the outcomes of package delivery delay when the four previously stated cooperative mechanisms are applied to the Prophet routing scheme. For 10 cooperative vehicles, the package delivery delay of CAPPS, HBDS, SACC, and IPS is 260%, 280%, 300%, and 320% respectively. It can be seen that the CAPPS technique outperforms the other strategies. The delivery delay of the CAPPS scheme is approximately 5%, 10%, and 15% lower than the HBDS, SACC, and IPS schemes respectively. Additionally, for 30 cooperative vehicles, the package delivery probability of CAPPS, HBDS, SACC, and IPS is 160%, 240%, 260%, and 270% respectively. Again it can be seen that the CAPPS technique outperforms the other strategies. The delivery delay of the CAPPS scheme is 15%, 17%, and 20% lower than HBDS, SACC, and IPS schemes respectively as shown in Fig 10.

Similarly, for 50 cooperative vehicles, the Delivery delay of CAPPS, HBDS, SACC, and IPS is 90%, 150%, 170%, and 230% respectively. Again the CAPPS technique outperforms all the compared techniques namely HBDS, SACC, and IPS schemes by around 15%, 20%, and 35% respectively. The CAPPS method outperforms all of the compared schemes, including HBDS, SACC, and IPS. The package delivery delay begins to reduce as the number of contact opportunities increases, as seen in Fig 10.

Fig 11 illustrates the outcomes for the package delivery delay when the four previously stated cooperative mechanisms are applied to the Spray and Wait routing scheme. For 10 cooperative vehicles, the package delivery delay of CAPPS, HBDS, SACC, and IPS is 230%, 260%, 260%, and 270% respectively. It can be seen that the CAPPS technique outperforms the other strategies. The delivery delay of the CAPPS scheme is approximately 7%, 7%, and 10% lower than the HBDS, SACC, and IPS schemes respectively. Additionally, for 30 cooperative vehicles, the package delivery probability of CAPPS, HBDS, SACC, and IPS is 150%, 180%, 200%, and 240% respectively. Again it can be seen that the CAPPS technique outperforms the other strategies. The delivery delay of the CAPPS scheme is 8%, 13%, and 23% lower than HBDS, SACC, and IPS schemes respectively. Similarly, for 50 cooperative vehicles, the Delivery delay of CAPPS, HBDS, SACC, and IPS is 80%, 130%, 170%, and 190% respectively. Again the CAPPS technique outperforms all the compared techniques namely HBDS, SACC, and IPS schemes by around 12%, 22%, and 27% respectively. The CAPPS method outperforms all of the compared schemes, including HBDS, SACC, and IPS. The package delivery delay begins to reduce as the number of interactions arises, as seen in Fig 11. Since nodes do not deviate from the standard, these findings indicate that forcing nodes to collaborate at a fixed percentage improves the performance of the network.

Figs 12 to 14 illustrates the outcomes for the energy consumption when the four previously stated cooperative mechanisms are applied to the Epidemic, ProPhet, and Spray and Wait routing protocols. Fig 12 illustrates the outcomes for the energy consumption when the four previously stated cooperative mechanisms are applied to the Epidemic routing scheme. For 10 cooperative vehicles, the energy consumption of CAPPS, HBDS, SACC, and IPS is 45 j, 50 j, 57 j, and 61 j respectively. It can be seen that the CAPPS technique outperforms the other strategies. The energy consumption of the CAPPS scheme is approximately 7%, 17%, and 24% lower than the HBDS, SACC, and IPS schemes respectively.

Additionally, for 30 cooperative vehicles, the energy consumption of CAPPS, HBDS, SACC, and IPS is 37 j, 47 j, 48 j, and 50 j respectively. Again it can be seen that the CAPPS technique outperforms the other strategies. The energy consumption of the CAPPS scheme is 14%, 17%, and 19% lower than HBDS, SACC, and IPS schemes respectively. Similarly, for 50 cooperative vehicles, the energy consumption of CAPPS, HBDS, SACC, and IPS is 18 j, 21 j, 23 j, and 25 j respectively. Again the CAPPS technique outperforms all the compared techniques namely HBDS, SACC, and IPS schemes by around 5%, 7%, and 10% respectively. The CAPPS method outperforms all of the compared schemes, including HBDS, SACC, and IPS. The energy consumption begins to reduce as the number of contact opportunities increases, as seen in Fig 12. The Energy consumption of CAPPS, HBDS, SACC, and IPS with Epidemic, Spray and Wait, and ProPhet routing protocol can be illustrated in Tables 9, 10, and 11 respectively.

**Table 9:** Energy consumption for CAPPS, HBDS, SACC, and IPS with Epidemic routing protocol according to the number of cooperative nodes.

No. of cooperative nodes	CAPPS	HBDS	SACC	IPS
10	45	50	57	61
20	37	42	45	49
30	35	39	43	46
40	32	36	38	40
50	15	17	18	19

**Table 10:** Energy consumption for CAPPS, HBDS, SACC, and IPS with Spray and Wait routing protocol according to the number of cooperative nodes.

No. of cooperative nodes	CAPPS	HBDS	SACC	IPS
10	30	39	43	46
20	26	29	33	40
30	18	25	31	38
40	19	24	26	29
50	10	13	14	16

**Table 11:** Energy Consumption for CAPPS, HBDS, SACC, and IPS with ProPhet routing protocol according to the number of cooperative nodes.

No. of cooperative nodes	CAPPS	HBDS	SACC	IPS
10	40	48	50	51
20	35	40	42	43
30	33	37	39	41
40	27	31	36	38
50	13	15	16	17

Fig 13 illustrates the outcomes for the energy consumption when the four previously stated cooperative mechanisms are applied to the ProPhet routing scheme. For 10 cooperative vehicles, the energy consumption of CAPPS, HBDS, SACC, and IPS is 39 j, 48 j, 49 j, and 52 j respectively. It can be seen that the CAPPS technique outperforms the other strategies. The energy consumption of the CAPPS scheme is approximately 13%, 15%, and 19% lower than the HBDS, SACC, and IPS schemes respectively. Additionally, for 30 cooperative vehicles, the energy consumption of CAPPS, HBDS, SACC, and IPS is 37 j, 43 j, 44 j, and 48 j respectively. Again it can be seen that the CAPPS technique outperforms the other strategies. The energy consumption of the CAPPS scheme is 9%, 10%, and 16% lower than HBDS, SACC, and IPS schemes respectively. Similarly, for 50 cooperative vehicles, the energy consumption of CAPPS, HBDS, SACC, and IPS is 13 j, 19 j, 20 j, and 22 j respectively. Again the CAPPS technique outperforms all the compared techniques namely HBDS, SACC, and IPS schemes by around 9%, 10%, and 13% respectively. The CAPPS method outperforms all of the compared schemes, including HBDS, SACC, and IPS. The energy consumption begins to reduce as the number of contact opportunities increases, as seen in Fig 13.

Fig 14 illustrates the outcomes for the energy consumption when the four previously stated cooperative mechanisms are applied to the Spray and Wait routing scheme. For 10 cooperative vehicles, the energy consumption of CAPPS, HBDS, SACC, and IPS is 29 j, 38 j, 44 j, and 46 j respectively. It can be seen that the CAPPS technique outperforms the other strategies. The energy consumption of the CAPPS scheme is approximately 13%, 21%, and 24% lower than the HBDS, SACC, and IPS schemes respectively. Additionally, for 30 cooperative vehicles, the energy consumption of CAPPS, HBDS, SACC, and IPS is 19 j, 26 j, 32 j, and 34 j respectively. Again it can be seen that the CAPPS technique outperforms the other strategies. The energy consumption of the CAPPS scheme is 10%, 19%, and 21.5% lower than HBDS, SACC, and IPS schemes respectively.

Similarly, for 50 cooperative vehicles, the energy consumption of CAPPS, HBDS, SACC, and IPS is 12 j, 18 j, 21 j, and 23 j respectively. Again the CAPPS technique outperforms all the compared techniques namely HBDS, SACC, and IPS schemes by around 8.5%, 13%, and 16% respectively. The CAPPS method outperforms all of the compared schemes, including HBDS, SACC, and IPS. The energy consumption begins to reduce as the number of contact opportunities increases, as seen in Fig 14. In this resource-restricted network environment, Spray-and-Wait outperforms both Epidemic and ProPhet routing protocols with greater delivery ratios while lower delay and energy consumption across all simulations, as shown in Figs 6 and 14. Because Spray-and-Wait reduces network transmissions by limiting the total number of copies contained in each package. This lowers some of the overhead associated with conventional epidemic diffusion.

## 7 Conclusion and future works

In the last few years, both the scientific community and the automotive industry have focused their research on vehicular designs. In this article, one of the major challenges of Congestion and selfishness faced by nodes in VDTN’s has been addressed. In VDTN’s, due to limited resources, the network’s nodes are unwilling to collaborate. These networks are always vulnerable to various disasters like fire and earthquakes due to the high volume of traffic. Because of their special features, which enable them to be used for communication and rescue/recovery operations during emergencies when other conventional network infrastructures are unavailable, VDTN’s are one way to handle these problems. Therefore, the Aware Payment and Punishment Scheme (CAPPS) for IoT-based VDTN’s networks is presented. Every vehicle in this scheme gives credit to the vehicle that shares its load. The loading managing framework manages congestion in a variety of scenarios, including low energy vehicles and congested vehicles as well as those in which both the vehicle and its neighbors are congested. The goal of CAPPS is to manage the overuse or underuse of vehicle resources and make appropriate use of the network resources. According to the simulation, CAPPS outperforms the other analyzed schemes in terms of energy consumption, delivery delay, and data delivery ratio.

Finally, a performance study was also conducted to assess the effect of the new techniques on the efficiency of VDTN’s using three distinct DTN routing protocols (Epidemic, ProPhet, and Spray and Wait). The CAPPS strategy (which requires vehicles to collaborate at almost the same percentage all of the time) outperforms the remaining of the recommended approaches. When using the Spray-and-Wait routing mechanism, all of the techniques produce better results. This research could be utilized to design a novel collaborative approach, like a cooperative strategy that pays vehicles for providing their resources to help other vehicles. Our future plans involve applying Machine Learning (ML) algorithms to predict the nodal workload and balance it accordingly. This avoidance mechanism aims to enhance link quality and optimize resource allocation. Additionally, leveraging node knowledge can minimize traffic congestion and improve load-sharing efficiency.

## References

[1] Ogbodo EU, Abu-Mahfouz AM, Kurien AM. A survey on 5G and LPWAN-IoT for improved smart cities and remote area applications: from the aspect of architecture and security. Sensors 2022;22(16):6313. doi: 10.3390/s22166313 36016078 PMC9412619

[2] Hassan R, Qamar F, Hasan MK, Aman AHM, Ahmed AS. Internet of things and its applications: a comprehensive survey. Symmetry 2020;12(10):1674. https://www.mdpi.com/2073-8994/12/10/1674#

[3] Khalil I, Khalil A, Ullah I, Tao Y, Khan I, Ashraf S et al. Social Internet of Things (SIoT): recent trends and its applications. In: Ullah I, Khan IU, Ouaissa M, El Hajjami S, Ouaissa M (eds) *Future Communication Systems Using Artificial Intelligence, Internet of Things and Data Science*. CRC Press 2024, p. 159–92.

[4] Sarker IH, Khan AI, Abushark YB, Alsolami F. Internet of things (IoT) security intelligence: a comprehensive overview, machine learning solutions and research directions. Mobile Netw Appl. 2023;28:296–312. doi: 10.1007/s11036-022-01937-3

[5] Badshah A, Ghani A, Daud A, Jalal A, Bilal M, Crowcroft J. Towards smart education through internet of things: a survey. ACM Comput Surv 2023;56(2):1–33. doi: 10.1145/3610401

[6] Fazel E, Najafabadi HE, Rezaei M, Leung H. Unlocking the power of mist computing through clustering techniques in IoT networks. Internet Things. 2023;22:100710.

[7] Alashjaee AM, Irshad A, Daud A, Alhomoud A, Altowaijri SM, Alshdadi AA. ReSOTS: RFID/IoT-enabled secure object tracking key exchange for trustworthy smart logistics. Wireless Personal Commun 2024;139(2):777–99

[8] Oladimeji D, Gupta K, Kose NA, Gundogan K, Ge L, Liang F. Smart transportation: an overview of technologies and applications. Sensors (Basel) 2023;23(8):3880. doi: 10.3390/s23083880 37112221 PMC10143476

[9] Ahmed A, Abdullah S, Iftikhar S, Ahmad I, Ajmal S, Hussain Q. A novel blockchain based secured and QoS aware IoT vehicular network in edge cloud computing. IEEE Access. 2022;10:77707–22. doi: 10.1109/ACCESS.2022.3192111

[10] Rehman G-U, Ghani A, Zubair M, Naqvi SHA, Singh D, Muhammad S. IPS: incentive and punishment scheme for omitting selfishness in the internet of vehicles (Iov). IEEE Access. 2019;7:109026–37. doi: 10.1109/ACCESS.2019.2921234

[11] Rehman GU, Ghani A, Zubair M, Ghayyure SA, Muhammad S. Honesty based democratic scheme to improve community cooperation for Internet of Things based vehicular delay tolerant networks. Trans Emerg Telecommun Technol. 2021;32(1):p.e4191. doi: 10.1002/ett.4191

[12] Ameur AI, Lakas A, Yagoubi MB, Oubbati OS. Peer-to-peer overlay techniques for vehicular ad hoc networks: survey and challenges. Veh Commun. 2022;34:100455. doi: 10.1016/j.vehcom.2022.100455

[13] Singh S, Anand V, Bera PK. A delay-tolerant low-duty cycle scheme in wireless sensor networks for IoT applications. Int J Cogn Comput Eng. 2023;204–204. doi: 10.1016/j.ijcce.2023.04.005

[14] Abdalla AM, Salamah SH. Performance comparison between delay-tolerant and non-delay-tolerant position-based routing protocols in VANETs. IJCNS 2022;15(1):1–14. doi: 10.4236/ijcns.2022.151001

[15] Liu E, Cao Y. Routing protocols in vehicular and aerial delay tolerant networks. In: Cao Y, Kaiwartya O, Li T (eds) Secure and Digitalized Future Mobility: Shaping the Ground and Air Vehicles Cooperation. CRC Press 2022, p. 155.

[16] Zhang B, Wang X, Xie R, Li C, Zhang H, Jiang F. A reputation mechanism based deep reinforcement learning and blockchain to suppress selfish node attack motivation in vehicular ad-hoc network. Future Gener Comput Syst. 2023;139:17–28.

[17] Chen M, Yi M, Huang M, Huang G, Ren Y, Liu A. A novel deep policy gradient action quantization for trusted collaborative computation in intelligent vehicle networks. Expert Syst Appl. 2023;221(C):119743. doi: 10.1016/j.eswa.2023.119743

[18] Chen J, Li T, Zhu R. Analysis of malicious node identification algorithm of internet of vehicles under blockchain technology: a case study of intelligent technology in automotive engineering. Appl Sci 2022;12(16):8362. doi: 10.3390/app12168362

[19] Azzedin F. Trust-based taxonomy for free riders in distributed multimedia systems. In: Proceedings of the 2010 International Conference on High Performance Computing & Simulation. HPCS 2010, June 28–July 2, 2010, Caen, France. IEEE 2010, p. 362–9. doi: 10.1109/hpcs.2010.5547108

[20] Rehman GU, Haq MIU, Zubair M, Mahmood Z, Singh M, Singh D. Misbehavior of nodes in IoT based vehicular delay tolerant networks VDTNs. Multimedia Tools and Applications 2023;82(5):7841–59. doi: 10.1007/s11042-022-13624-2

[21] Liu Y, Wang K, Guo H, Lu Q, Sun Y. Social-aware computing based congestion control in delay tolerant networks. Mobile Netw Appl 2016;22(2):174–85. doi: 10.1007/s11036-016-0759-8

[22] Jiang Q, Men C, Tian Z. A credit-based congestion-aware incentive scheme for DTNs. Information 2016;7(4):71. doi: 10.3390/info7040071

[23] Kou M, Zhao Y, Cai H, Fan X. Study of a routing algorithm of Internet of vehicles based on selfishness. In: 2018 IEEE International Conference on Smart Internet of Things (SmartIoT), Xián, 2018, p. 34–-39. doi: 10.1109/SmartIoT.2018.00016

[24] Aliedani A, Loke SW, Glaser S. Robust cooperative car-parking: implications and solutions for selfish inter-vehicular social behaviour. Human-Centric Comput Inf Sci 2020;10(1):1–28. doi: 10.1186/s13673-020-00243-9

[25] Rehman GU, Zubair M, Qasim I, Badshah A, Mahmood Z, Aslam M, et al. EMS: efficient monitoring system to detect non-cooperative nodes in IoT-based vehicular delay tolerant networks (VDTNs). Sensors (Basel) 2022;23(1):99. doi: 10.3390/s23010099 36616697 PMC9824832

[26] Sharma R, Gupta DV. A reputation-based mechanism to detect selfish nodes in DTNs. In: Kumar A, Mozar S (eds) Proceedings of the International Conference on Communications and Cyber Physical Engineering. Lecture Notes in Electrical Engineering, vol. 500. Singapore: Springer. 2018:55–61. doi: 10.1007/978-981-13-0212-1

[27] Socievole A, Caputo A, De Rango F, Fazio P. Routing in mobile opportunistic social networks with selfish nodes. Wirel. Commun. Mob. Comput. 2019;2019:1–15. doi: 10.1155/2019/6359806

[28] Xia Z, Mao X, Gu K, Jia W. Two-dimensional behavior-marker-based data forwarding incentive scheme for fog-computing-based SIoVs. IEEE Trans Comput Soc Syst 2021;5(1):1406–18. doi: 10.1109/TCSS.2021.3129898

[29] Rehman GU, Ghani A, Zubair M, Saeed MI, Singh D. SOS: Socially omitting selfishness in IoT for smart and connected communities. Int J Commun Syst 2023;36(1):e4455. doi: 10.48550/arXiv.2004.08948

[30] Rehman GU, Haq MIU, Badshah A, Zubair M, Daraz A, AlQahtani SA, et al. Reliable community card system for detecting and isolating selfish vehicles in smart and connected communities-based VDTN’s. Trans. Emerg. Telecommun. Technol 2024;35(6):e5006. doi: 10.1002/ett.5006

[31] Sharma R, Dinkar SK. Selfish node detection by modularized deep NMF autoencoder based incentivized reputation scheme. Cybern Syst. 2023;54(7):1172–-1198.

[32] Rajput NS, Banerjee R, Sanghi D, Kalyansundaram C. Evolution of cooperation in vehicular cloud assisted networks for ITS services: a hunt game-based approach. Future Gener Comput Syst 2023;146(6):62–77. doi: 10.1016/j.future.2023.04.013

[33] Jaramillo JJ, Srikant R. A game theory based reputation mechanism to incentivize cooperation in wireless ad hoc networks. Ad Hoc Netw. 2010;8(4):416–29. doi: 10.1016/j.adhoc.2009.10.002

[34] Kanmani S, Murali M. Computation of PoA for selfish node detection and resource allocation using game theory. Comput Syst Sci Eng. 2023;47(2):2583–98. doi: 10.32604/csse.2023.037265

[35] Nobahary S, Garakani HG, Khademzadeh A, Rahmani AM. Selfish node detection based on hierarchical game theory in IoT. J Wireless Commun Network 2019;2019(1):1–19. doi: 10.1186/s13638-019-1564-4

[36] Sharah A, Alhaj M, Hassan M. Selfish dynamic punishment scheme: misbehavior detection in MANETs using cooperative repeated game. IJCSNS. 2020;20(33):168–73

[37] Mostefa FZ, Maaza ZM, Duvallet C. Secure communications by tit-for-tat strategy in vehicular networks. Int J Networked Distributed Comput 2020;8(4):214–21. doi: 10.2991/ijndc.k.200925.001

[38] Park Y, Sur C, Rhee K-H. A secure incentive scheme for vehicular delay tolerant networks using cryptocurrency. Security Commun Netw. 2018;2018:1–13. doi: 10.1155/2018/5932183

[39] Kumar S, Dutta K. Trust based intrusion detection technique to detect selfish nodes in mobile ad hoc networks. Wireless Pers Commun 2018;101(4):2029–52. doi: 10.1007/s11277-018-5804-4

[40] Nabais C, Pereira PR, Magaia N. BiRep: a reputation scheme to mitigate the effects of black-hole nodes in delay-tolerant internet of vehicles. Sensors (Basel) 2021;21(3):835. doi: 10.3390/s21030835 33513736 PMC7866179

[41] Rosas E, Garay F, Hidalgo N. Context-aware self-adaptive routing for delay tolerant network in disaster scenarios. Ad Hoc Netw. 2020;102:102095. doi: 10.1016/j.adhoc.2020.102095

[42] Keykhaie S, Rostaei M. Congestion- and selfishness-aware social routing in delay tolerant networks. In: 2017 7th International Conference on Computer and Knowledge Engineering (ICCKE). 2017, p. 439–44. doi: 10.1109/iccke.2017.8167918

[43] Abdi GH, Sheikhani AHR, Kordrostami S, Ghane A, Babaie S. A novel selfish node detection based on reputation and game theory in internet of things. Computing 2023;106(1):81–107. doi: 10.1007/s00607-023-01184-8

[44] Soares VNGJ, Farahmand F, Rodrigues JJPC. VDTNsim: a simulation tool for vehicular delay-tolerant networks. In: 2010 15th IEEE International Workshop on Computer Aided Modeling, Analysis and Design of Communication Links and Networks (CAMAD). 2010, p. 101–5. doi: 10.1109/camad.2010.5686980

[45] Zhang X, Neglia G, Kurose J, Towsley D. Performance modeling of epidemic routing. Comput Netw 2007;51(10):2867–91. doi: 10.1016/j.comnet.2006.11.028

[46] Phearin Sok, Keecheon Kim. Distance-based PRoPHET routing protocol in disruption tolerant network. In: 2013 International Conference on ICT Convergence (ICTC). 2013, p. 159–64. doi: 10.1109/ictc.2013.6675329

[47] Wang E, Yang Y, Wu J, Liu W. A buffer management strategy on spray and wait routing protocol in DTNs. In: 2015 44th International Conference on Parallel Processing. 2015, p. 799–808. doi: 10.1109/icpp.2015.89

